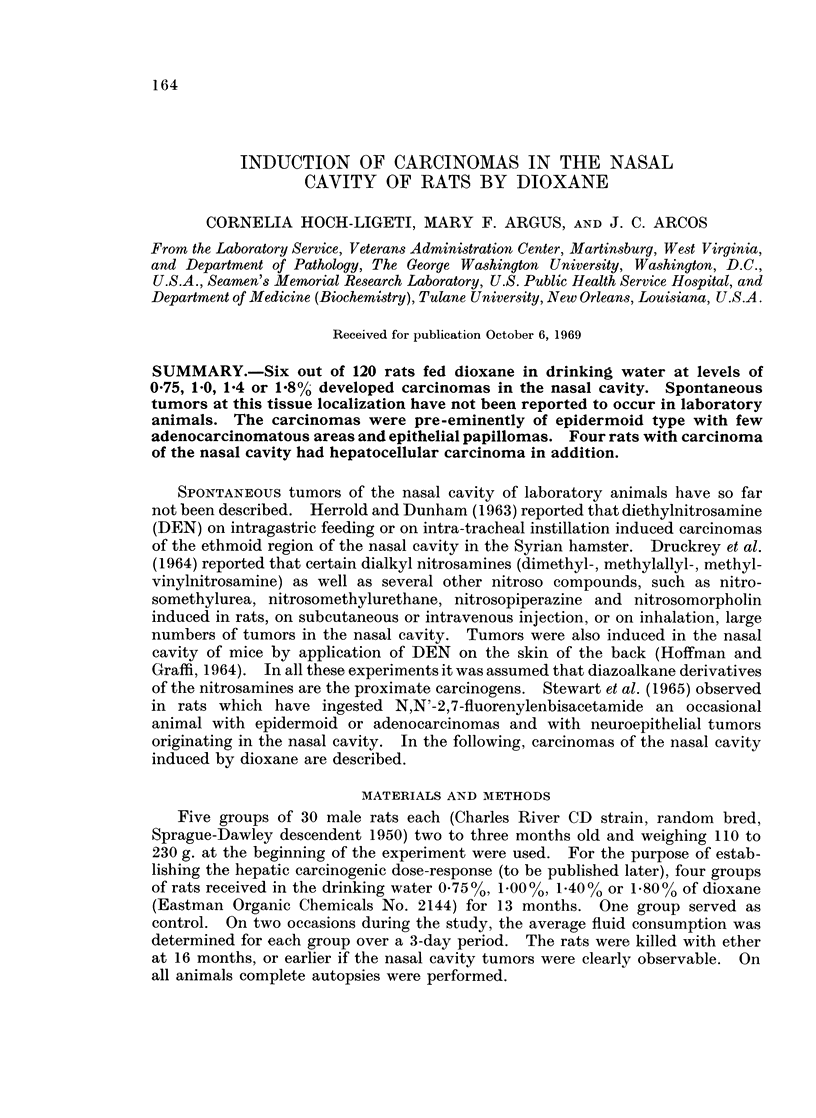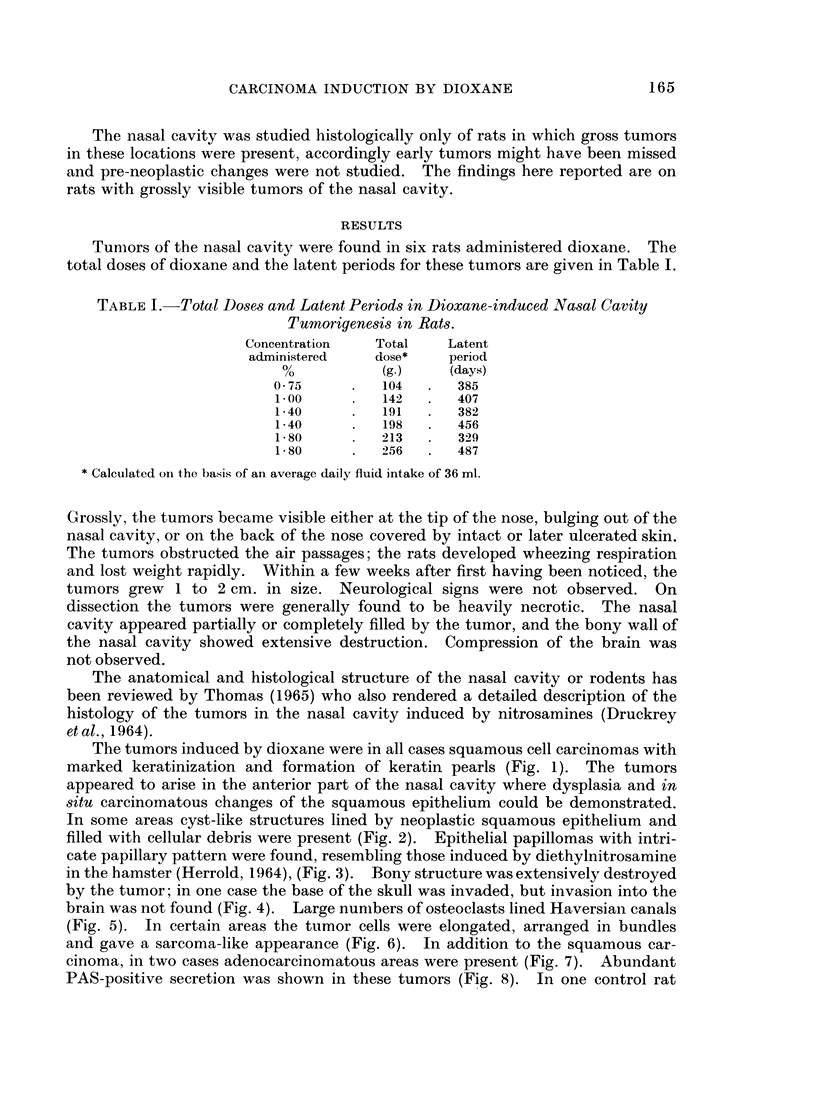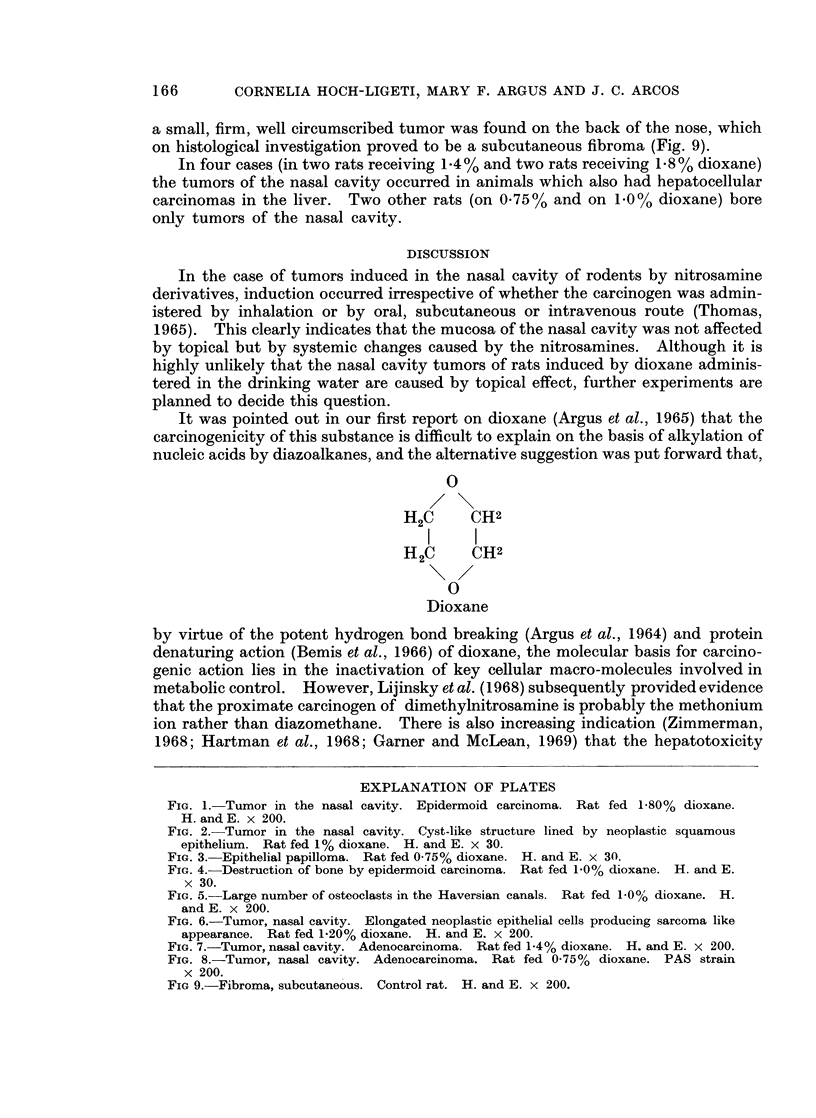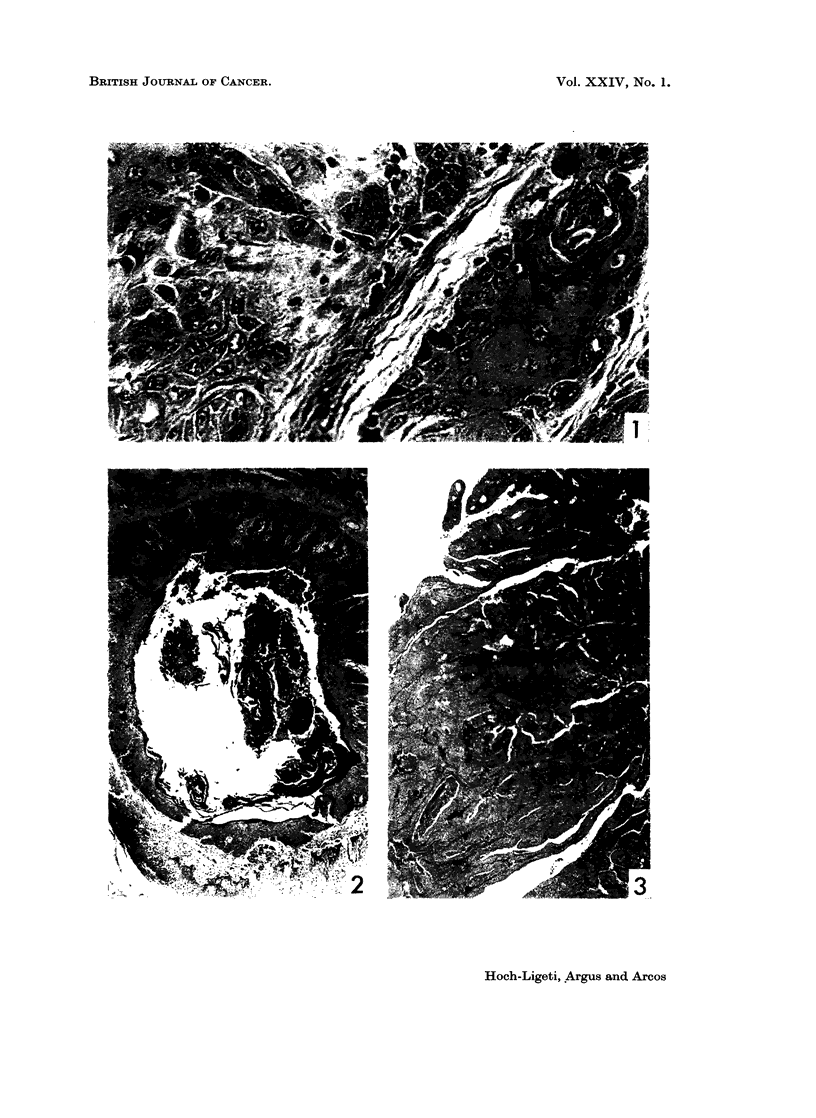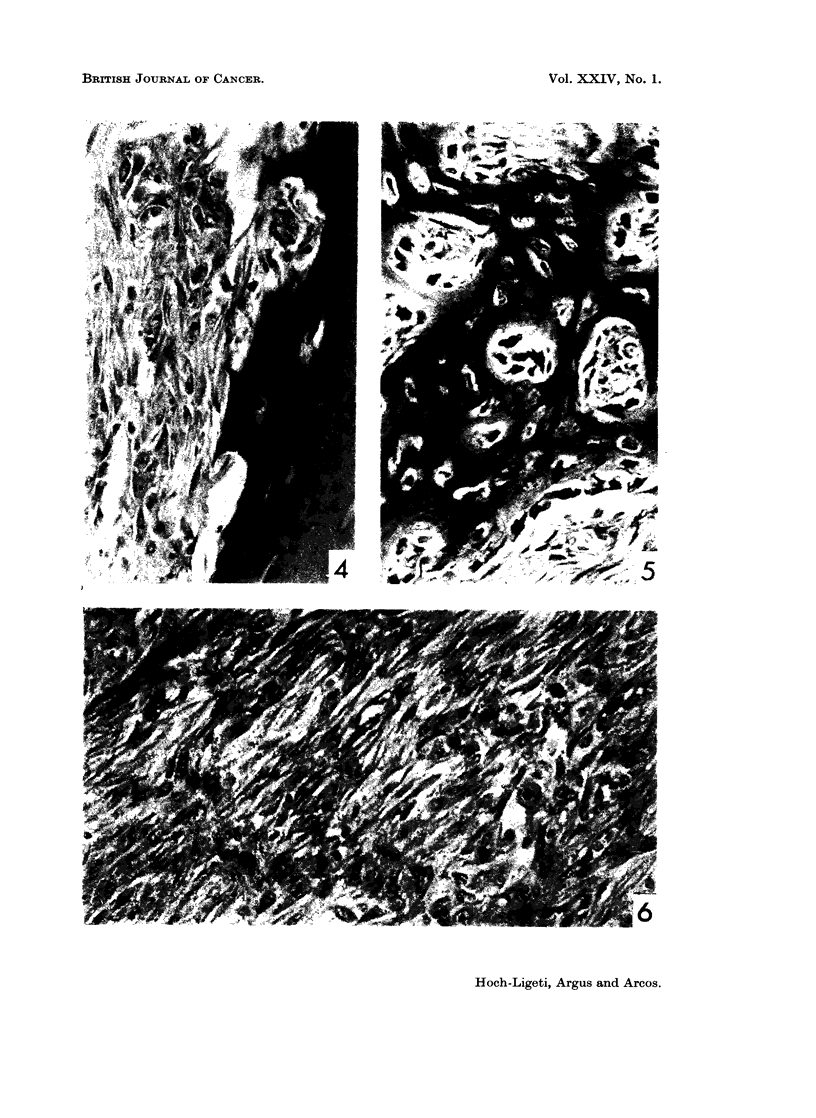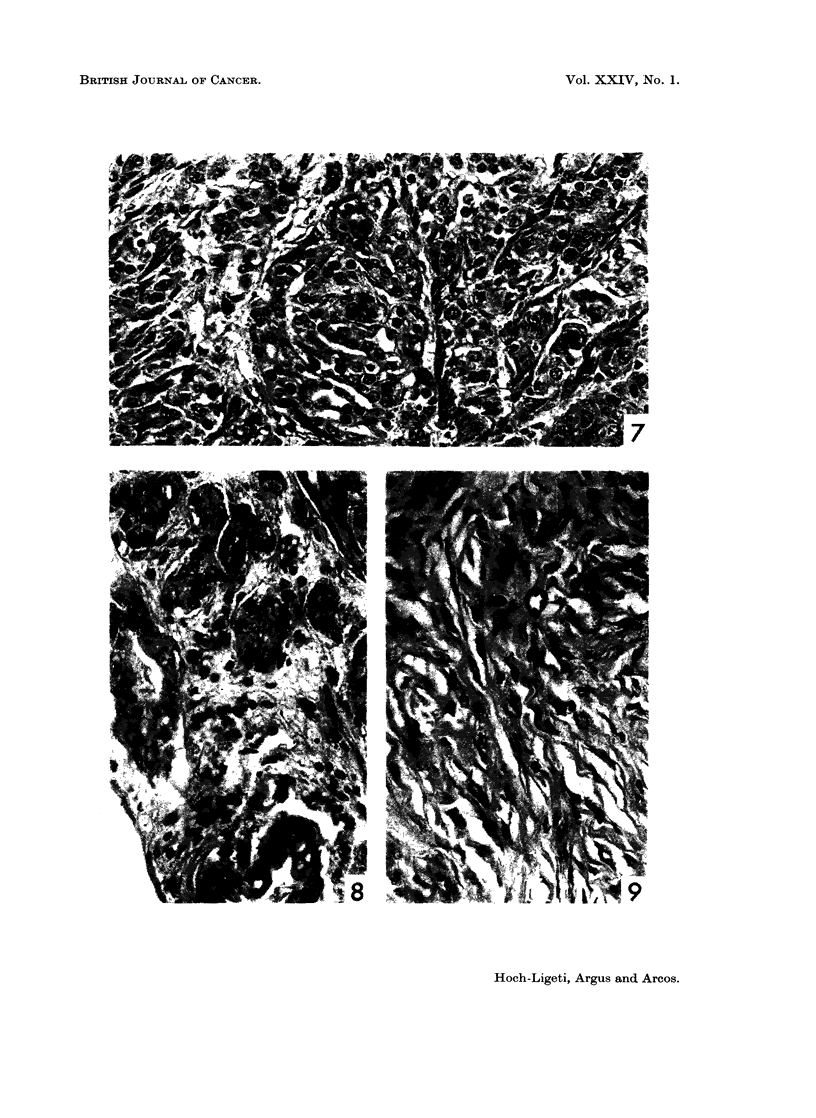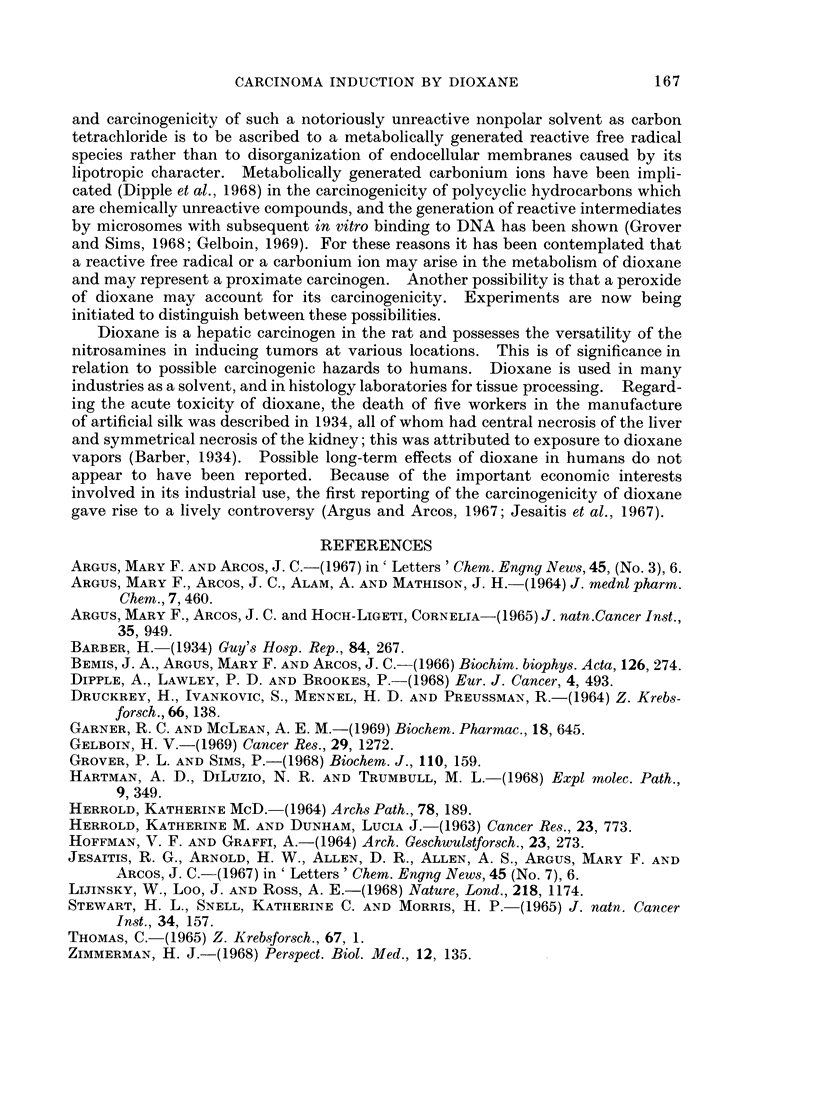# Induction of Carcinomas in the Nasal Cavity of Rats by Dioxane

**DOI:** 10.1038/bjc.1970.19

**Published:** 1970-03

**Authors:** Cornelia Hoch-Ligeti, Mary F. Argus, J. C. Arcos

## Abstract

**Images:**


### INDUCTION OF CARCINOMAS IN THE NASAL CAVITY OF RATS BY DIOXANE

CORNELIA HOCH-LIGETI, MARY F. ARGUS, AND J. C. ARCOS

*From the Laboratory Service, Veterans Administration Center, Martinsburg, West Virginia, and Department of Pathology, The George Washington University, Washington, D.C., U.S.A., Seamen's Memorial Research Laboratory, U.S. Public Health Service Hospital, and Department of Medicine (Biochemistry), Tulane University, New Orleans, Louisiana, U.S.A.*

Received for publication October 6, 1969

**SUMMARY.—Six out of 120 rats fed dioxane in drinking water at levels of 0·75, 1·0, 1·4 or 1·8% developed carcinomas in the nasal cavity. Spontaneous tumors at this tissue localization have not been reported to occur in laboratory animals. The carcinomas were pre-eminently of epidermoid type with few adenocarcinomatous areas and epithelial papillomas. Four rats with carcinoma of the nasal cavity had hepatocellular carcinoma in addition.**

SPONTANEOUS tumors of the nasal cavity of laboratory animals have so far not been described. Herrold and Dunham (1963) reported that diethylnitrosamine (DEN) on intragastric feeding or on intra-tracheal instillation induced carcinomas of the ethmoid region of the nasal cavity in the Syrian hamster. Druckrey *et al.* (1964) reported that certain dialkyl nitrosamines (dimethyl-, methylallyl-, methylvinylnitrosamine) as well as several other nitroso compounds, such as nitrosomethylurea, nitrosomethylurethane, nitrosopiperazine and nitrosomorpholin induced in rats, on subcutaneous or intravenous injection, or on inhalation, large numbers of tumors in the nasal cavity. Tumors were also induced in the nasal cavity of mice by application of DEN on the skin of the back (Hoffman and Graffi, 1964). In all these experiments it was assumed that diazoalkane derivatives of the nitrosamines are the proximate carcinogens. Stewart *et al.* (1965) observed in rats which have ingested N,N'-2,7-fluorenylenbisacetamide an occasional animal with epidermoid or adenocarcinomas and with neuroepithelial tumors originating in the nasal cavity. In the following, carcinomas of the nasal cavity induced by dioxane are described.

### MATERIALS AND METHODS

Five groups of 30 male rats each (Charles River CD strain, random bred, Sprague-Dawley descendent 1950) two to three months old and weighing 110 to 230 g. at the beginning of the experiment were used. For the purpose of establishing the hepatic carcinogenic dose-response (to be published later), four groups of rats received in the drinking water 0·75%, 1·00%, 1·40% or 1·80% of dioxane (Eastman Organic Chemicals No. 2144) for 13 months. One group served as control. On two occasions during the study, the average fluid consumption was determined for each group over a 3-day period. The rats were killed with ether at 16 months, or earlier if the nasal cavity tumors were clearly observable. On all animals complete autopsies were performed.

The nasal cavity was studied histologically only of rats in which gross tumors in these locations were present, accordingly early tumors might have been missed and pre-neoplastic changes were not studied. The findings here reported are on rats with grossly visible tumors of the nasal cavity.

### RESULTS

Tumors of the nasal cavity were found in six rats administered dioxane. The total doses of dioxane and the latent periods for these tumors are given in Table I.

TABLE I.—*Total Doses and Latent Periods in Dioxane-induced Nasal Cavity Tumorigenesis in Rats.*

| Concentration administered % | Total dose* (g.) | Latent period (days) |
|---|---|---|
| 0·75 | 104 | 385 |
| 1·00 | 142 | 407 |
| 1·40 | 191 | 382 |
| 1·40 | 198 | 456 |
| 1·80 | 213 | 329 |
| 1·80 | 256 | 487 |

* Calculated on the basis of an average daily fluid intake of 36 ml.

Grossly, the tumors became visible either at the tip of the nose, bulging out of the nasal cavity, or on the back of the nose covered by intact or later ulcerated skin. The tumors obstructed the air passages; the rats developed wheezing respiration and lost weight rapidly. Within a few weeks after first having been noticed, the tumors grew 1 to 2 cm. in size. Neurological signs were not observed. On dissection the tumors were generally found to be heavily necrotic. The nasal cavity appeared partially or completely filled by the tumor, and the bony wall of the nasal cavity showed extensive destruction. Compression of the brain was not observed.

The anatomical and histological structure of the nasal cavity or rodents has been reviewed by Thomas (1965) who also rendered a detailed description of the histology of the tumors in the nasal cavity induced by nitrosamines (Druckrey *et al.*, 1964).

The tumors induced by dioxane were in all cases squamous cell carcinomas with marked keratinization and formation of keratin pearls (Fig. 1). The tumors appeared to arise in the anterior part of the nasal cavity where dysplasia and *in situ* carcinomatous changes of the squamous epithelium could be demonstrated. In some areas cyst-like structures lined by neoplastic squamous epithelium and filled with cellular debris were present (Fig. 2). Epithelial papillomas with intricate papillary pattern were found, resembling those induced by diethylnitrosamine in the hamster (Herrold, 1964), (Fig. 3). Bony structure was extensively destroyed by the tumor; in one case the base of the skull was invaded, but invasion into the brain was not found (Fig. 4). Large numbers of osteoclasts lined Haversian canals (Fig. 5). In certain areas the tumor cells were elongated, arranged in bundles and gave a sarcoma-like appearance (Fig. 6). In addition to the squamous carcinoma, in two cases adenocarcinomatous areas were present (Fig. 7). Abundant PAS-positive secretion was shown in these tumors (Fig. 8). In one control rat a small, firm, well circumscribed tumor was found on the back of the nose, which on histological investigation proved to be a subcutaneous fibroma (Fig. 9).

In four cases (in two rats receiving 1·4% and two rats receiving 1·8% dioxane) the tumors of the nasal cavity occurred in animals which also had hepatocellular carcinomas in the liver. Two other rats (on 0·75% and on 1·0% dioxane) bore only tumors of the nasal cavity.

### DISCUSSION

In the case of tumors induced in the nasal cavity of rodents by nitrosamine derivatives, induction occurred irrespective of whether the carcinogen was administered by inhalation or by oral, subcutaneous or intravenous route (Thomas, 1965). This clearly indicates that the mucosa of the nasal cavity was not affected by topical but by systemic changes caused by the nitrosamines. Although it is highly unlikely that the nasal cavity tumors of rats induced by dioxane administered in the drinking water are caused by topical effect, further experiments are planned to decide this question.

It was pointed out in our first report on dioxane (Argus *et al.*, 1965) that the carcinogenicity of this substance is difficult to explain on the basis of alkylation of nucleic acids by diazoalkanes, and the alternative suggestion was put forward that,

Dioxane by virtue of the potent hydrogen bond breaking (Argus *et al.*, 1964) and protein denaturing action (Bemis *et al.*, 1966) of dioxane, the molecular basis for carcinogenic action lies in the inactivation of key cellular macro-molecules involved in metabolic control. However, Lijinsky *et al.* (1968) subsequently provided evidence that the proximate carcinogen of dimethylnitrosamine is probably the methonium ion rather than diazomethane. There is also increasing indication (Zimmerman, 1968; Hartman *et al.*, 1968; Garner and McLean, 1969) that the hepatotoxicity

---

### EXPLANATION OF PLATES

FIG. 1.—Tumor in the nasal cavity. Epidermoid carcinoma. Rat fed 1·80% dioxane. H. and E. × 200.
FIG. 2.—Tumor in the nasal cavity. Cyst-like structure lined by neoplastic squamous epithelium. Rat fed 1% dioxane. H. and E. × 30.
FIG. 3.—Epithelial papilloma. Rat fed 0·75% dioxane. H. and E. × 30.
FIG. 4.—Destruction of bone by epidermoid carcinoma. Rat fed 1·0% dioxane. H. and E. × 30.
FIG. 5.—Large number of osteoclasts in the Haversian canals. Rat fed 1·0% dioxane. H. and E. × 200.
FIG. 6.—Tumor, nasal cavity. Elongated neoplastic epithelial cells producing sarcoma like appearance. Rat fed 1·20% dioxane. H. and E. × 200.
FIG. 7.—Tumor, nasal cavity. Adenocarcinoma. Rat fed 1·4% dioxane. H. and E. × 200.
FIG. 8.—Tumor, nasal cavity. Adenocarcinoma. Rat fed 0·75% dioxane. PAS strain × 200.
FIG 9.—Fibroma, subcutaneous. Control rat. H. and E. × 200.

1

2

3

Hoch-Ligeti, Argus and Arcos

Hoch-Ligeti, Argus and Arcos.

Hoch-Ligeti, Argus and Arcos.

and carcinogenicity of such a notoriously unreactive nonpolar solvent as carbon tetrachloride is to be ascribed to a metabolically generated reactive free radical species rather than to disorganization of endocellular membranes caused by its lipotropic character. Metabolically generated carbonium ions have been implicated (Dipple *et al.*, 1968) in the carcinogenicity of polycyclic hydrocarbons which are chemically unreactive compounds, and the generation of reactive intermediates by microsomes with subsequent *in vitro* binding to DNA has been shown (Grover and Sims, 1968; Gelboin, 1969). For these reasons it has been contemplated that a reactive free radical or a carbonium ion may arise in the metabolism of dioxane and may represent a proximate carcinogen. Another possibility is that a peroxide of dioxane may account for its carcinogenicity. Experiments are now being initiated to distinguish between these possibilities.

Dioxane is a hepatic carcinogen in the rat and possesses the versatility of the nitrosamines in inducing tumors at various locations. This is of significance in relation to possible carcinogenic hazards to humans. Dioxane is used in many industries as a solvent, and in histology laboratories for tissue processing. Regarding the acute toxicity of dioxane, the death of five workers in the manufacture of artificial silk was described in 1934, all of whom had central necrosis of the liver and symmetrical necrosis of the kidney; this was attributed to exposure to dioxane vapors (Barber, 1934). Possible long-term effects of dioxane in humans do not appear to have been reported. Because of the important economic interests involved in its industrial use, the first reporting of the carcinogenicity of dioxane gave rise to a lively controversy (Argus and Arcos, 1967; Jesaitis *et al.*, 1967).

### REFERENCES

ARGUS, MARY F. AND ARCOS, J. C.—(1967) in ‘ Letters ’ *Chem. Engng News*, **45**, (No. 3), 6.

ARGUS, MARY F., ARCOS, J. C., ALAM, A. AND MATHISON, J. H.—(1964) *J. mednl pharm. Chem.*, **7**, 460.

ARGUS, MARY F., ARCOS, J. C. and HOCH-LIGETI, CORNELIA—(1965) *J. natn.Cancer Inst.*, **35**, 949.

BARBER, H.—(1934) *Guy's Hosp. Rep.*, **84**, 267.

BEMIS, J. A., ARGUS, MARY F. AND ARCOS, J. C.—(1966) *Biochim. biophys. Acta*, **126**, 274.

DIPPLE, A., LAWLEY, P. D. AND BROOKES, P.—(1968) *Eur. J. Cancer*, **4**, 493.

DRUCKREY, H., IVANKOVIC, S., MENNEL, H. D. AND PREUSSMAN, R.—(1964) *Z. Krebsforsch.*, **66**, 138.

GARNER, R. C. AND MCLEAN, A. E. M.—(1969) *Biochem. Pharmac.*, **18**, 645.

GELBOIN, H. V.—(1969) *Cancer Res.*, **29**, 1272.

GROVER, P. L. AND SIMS, P.—(1968) *Biochem. J.*, **110**, 159.

HARTMAN, A. D., DILUZIO, N. R. AND TRUMBULL, M. L.—(1968) *Expl molec. Path.*, **9**, 349.

HERROLD, KATHERINE MCD.—(1964) *Archs Path.*, **78**, 189.

HERROLD, KATHERINE M. AND DUNHAM, LUCIA J.—(1963) *Cancer Res.*, **23**, 773.

HOFFMAN, V. F. AND GRAFFI, A.—(1964) *Arch. Geschwulstforsch.*, **23**, 273.

JESAITIS, R. G., ARNOLD, H. W., ALLEN, D. R., ALLEN, A. S., ARGUS, MARY F. AND ARCOS, J. C.—(1967) in ‘ Letters ’ *Chem. Engng News*, **45** (No. 7), 6.

LIJINSKY, W., LOO, J. AND ROSS, A. E.—(1968) *Nature, Lond.*, **218**, 1174.

STEWART, H. L., SNELL, KATHERINE C. AND MORRIS, H. P.—(1965) *J. natn. Cancer Inst.*, **34**, 157.

THOMAS, C.—(1965) *Z. Krebsforsch.*, **67**, 1.

ZIMMERMAN, H. J.—(1968) *Perspect. Biol. Med.*, **12**, 135.